# Relationship between Aspartame-Induced Cerebral Cortex Injury and Oxidative Stress, Inflammation, Mitochondrial Dysfunction, and Apoptosis in Sprague Dawley Rats

**DOI:** 10.3390/antiox13010002

**Published:** 2023-12-19

**Authors:** Jureeporn U-pathi, Yen-Chia Yeh, Chia-Wen Chen, Eddy E. Owaga, Rong-Hong Hsieh

**Affiliations:** 1 School of Nutrition and Health Sciences, College of Nutrition, Taipei Medical University, Taipei 11031, Taiwan; da07108007@tmu.edu.tw (J.U.-p.); ma07108006@tmu.edu.tw (Y.-C.Y.); 2 Research Center of Nutritional Medicine, College of Nutrition, Taipei Medical University, Taipei 11031, Taiwan; d301091008@tmu.edu.tw; 3 Institute of Food Bioresources Technology, Dedan Kimathi University of Technology, Nyeri P.O. Box 657-10100, Kenya; eddy.owaga@dkut.ac.ke; 4 Ph.D. Program in Drug Discovery and Development Industry, College of Pharmacy, Taipei Medical University, Taipei 11031, Taiwan

**Keywords:** aspartame, inflammation, oxidative stress, mitochondrial biogenesis, apoptosis

## Abstract

There are emerging concerns about the potential cerebral cortex injury from aspartame due to the accumulation of the various neurotoxic metabolic components in the central nervous system after long-term dietary exposure. The aim of this study was to evaluate the effect of oral aspartame consumption on cerebral cortex injury in the rat brain, and further evaluate the various underlying molecular mechanisms, with a special focus on oxidative stress, inflammation, mitochondrial dysfunction, and apoptosis pathways. Sprague Dawley rats (nineteen, female) were randomly sub-divided into three groups: (i) normal diet with vehicle: control group (five rats), (ii) low dose of aspartame group (LA): seven rats received 30 mg/kg body weight (bw) daily doses of aspartame, (iii) high dose of aspartame group (HA): seven rats received 60 mg/kg bw daily doses of aspartame. After 8 weeks, the LA and HA groups showed lower expression levels of brain-derived neurotrophic factor (BDNF), antioxidant enzyme activity (SOD2, CAT), antioxidant marker (Nrf2), inflammatory response (IκB), mitochondrial biogenesis (Sirt1, PGC1α, Nrf1, TFAM), mitochondrial DNA (mtDNA) copy number, and apoptosis-related proteins (Bax, Caspase-3) expressions. Aspartame administration also elevated oxidative stress levels (Malondialdehyde, MDA), 8-hydroxy-2-deoxy guanosine (8-OHdG), PGE_2_ and COX-2 expressions, pro-inflammatory cytokines (TNFα, IL6, IL1β), antioxidant marker expression (Keap1), inflammatory responses (iNOS, NFκB), and glial fibrillary acidic protein (GFAP) levels in the cerebral cortex of the rats, thereby contributing to the reduced survival of pyramidal cells and astrocyte glial cells of the cerebral cortex. Therefore, these findings imply that aspartame-induced neurotoxicity in rats’ cerebral cortex could be regulated through four mechanisms: inflammation, enhanced oxidant stress, decreased mitochondrial biogenesis, and apoptosis pathways.

## 1. Introduction

Aspartame has been widely used in many food products as an artificial sweetener for over a century. Although aspartame is about 180–200 times sweeter than sucrose, it contains less caloric value [1]. Aspartame is a dipeptide of aspartic acid and phenylalanine amino acids and can be broken down in the intestinal lumen into phenylalanine (40%), methanol (10%), and aspartic acid (50%) upon consumption. These secondary metabolites can further be converted into formaldehyde and formic acid, which have been associated with various neurochemical effects [2,3,4]. Phenylalanine is essential for the synthesis of neurotransmitters, including dihydroxyphenylalanine, epinephrine, nor-epinephrine, and phenylethylamine, whereas aspartic acid plays a crucial role as an excitatory neurotransmitter [5]. Methanol can be converted into formate in the body and further excreted or converted to highly neurotoxic compounds, which have been associated with several neurological and behavioral disturbances [3].

Aspartame is commonly used as a sweetener in chewing gum, yogurts, supplements, desserts, medicines, and diet beverages [6]. Millions of people worldwide consume aspartame, including diabetic patients, children, and pregnant women [2]. However, there are emerging concerns about the potential neurotoxic effects of aspartame due to the accumulation of the various neurotoxic metabolic components in the central nervous system after long-term dietary exposure [2,3,4]. Furthermore, there are controversies over the safety of some sweeteners, including some reports indicating aspartame may affect fertility by reserving fewer follicles in the ovary and disrupting steroidogenesis in granulosa cells. Most of the previous studies have attempted to explain aspartame-induced brain damage; however, the potential association between aspartame-induced oxidative stress, mitochondrial dysfunction, inflammation, and apoptosis pathways in cerebral cortex injury in rats has not been elucidated.

Therefore, the purpose of this study was to investigate the influence of oral aspartame consumption on cerebral cortex injury in the rat brain and further evaluate the potential role of various underlying molecular mechanisms, with a special focus on oxidative stress, inflammation, mitochondrial dysfunction, and apoptosis pathways.

## 2. Materials and Methods

### 2.1. Materials

Aspartame (98%) was purchased from Alfa Aesar (Lancashire, UK).

### 2.2. Animals and Experimental Procedures

Female Sprague Dawley rats (7 weeks old; weighing 120–140 g) were used in this study. The rats were collected from National Laboratory Animal Center (Taipei, Taiwan) and were acclimatized in a room under controlled temperature and humidity for 1 week. All rats were fed with Laboratory rodent diet 5001 (57% carbohydrate, 13% fat, 30% protein) and water ad libitum for 8 weeks. The rats were then randomly divided into three groups: (i) normal diet with vehicle: control group (5 rats), (ii) low-dose of aspartame group (LA): 7 rats received 30 mg/kg body weight (bw) daily doses of aspartame in relation to the acceptable daily intake (ADI), (iii) high-dose of aspartame group (HA): 7 rats received 60 mg/kg bw daily doses of aspartame in relation to the ADI. ADI was calculated according to the human ADI guidelines [6]. The individual rats in each group were weighed at the start and end of the experiment. After 8 weeks of feeding with respective diets, the rats were anesthetized with isoflurane gas, and the whole brain was removed and weighed. The brain coefficient for each rat was calculated as brain weight/body weight. Thereafter, brain samples were kept at −80 °C awaiting further analysis. The animal study protocol was approved by the Institutional Animal Care and Use Committee of Taipei Medical University (IACUC Approval No: LAC-2020-0176, LAC-2020-0278).

### 2.3. Measurement of Brain Function Biomarkers

The brain components were removed, and the cerebrum sections were dissected, blotted, dried, weighed, and placed on ice. They were then homogenized and centrifuged at 5000 rpm (4 °C) for 15 min, and the total protein contents of supernatant determined using Pierce^®^ BCA protein assay kit (Thermo Fisher Scientific, Waltham, MA, USA). Thereafter, the following brain function biomarkers were analyzed: brain-derived neurotrophic factor (BDNF) using the R&D systems ELISA kit (Minneapolis, MN, USA); Cyclooxygenases-2 (COX-2) and Prostanoids (PGE_2_) using Cusabio ELISA kit (Houston, TX, USA) and Cayman Chemical ELISA kit (Ann Arbor, Miami, FL, USA), respectively.

### 2.4. Measurement of Glutathione Peroxidase (GPx), 8-Hydroxy-2-deoxy Guanosine (8-OHdG), and Malondialdehyde (MDA) in Rat Brain

The GPx activity was determined by the evaluation of its ability to catalyze standard glutathione (GSH) in the presence of hydrogen peroxide (1 mM H_2_O_2_) using Cayman Chemical assay kit (Ann Arbor, MI, USA). The expression of 8-OHdG was assayed using Cusabio ELISA kit (Houston, TX, USA) according to the manufacturer’s protocol. Total protein content of tissue samples was analyzed using Pierce^®^ BCA protein assay kit (Thermo Fisher Scientific, USA). Malondialdehyde (MDA), a by-product of lipid peroxidation, was determined by thiobarbituric acid (TBARS) modified method of Chang et al. [7]. A combination of 20 µL of brain homogenate, 50 µL of 30 g/L sodium dodecyl sulfate, 200 µL of 0.1 M HCl, 30 µL of 10 g/L phosphotungstic acid, and 100 µL of 7 g/L 2-thiobarbituric acid were incubated in boiling water for 30 min. After cooling, 500 µL of 1-butanol was added. The mixture was centrifuged at 1000× *g* for 10 min at 4 °C before collection of the organic layer. The absorbance was measured at 532 nm and compared with a standard curve of known concentrations of 1, 1,3,3-tetramethoxypropane. The data were expressed as nanomoles MDA per milligram protein.

### 2.5. Histological Preparation and Examination

Nine coronal sections of the rat brain from the control and treatment groups were used for histology. The target sections for morphological assays were fixed in 10% formaldehyde at room temperature, and cryoprotected in 10% and 20% sucrose (1 h) and then 30% sucrose (24 h) at room temperature. The samples were then immediately frozen with an optimal cutting temperature (OCT) compound (Sakura Finetek Japan, Tokyo, Japan) and kept at −80 °C until analysis. The brain components were serially sliced into coronal sections (25 μm) using cryostat (CM3050S, Leica Biosystems, Nussloch, Germany), then placed onto gelatin-coated microscope slides (Southern Biotech, Birmingham, AL, USA) and air-dried overnight. The coronal sections were stained with hematoxylin and eosin (H&E) for general histology procedures. The appearance of the six layers of the cerebral cortex was characterized as follows: external granular layer, outer molecular layer, external pyramidal layer, inner pyramidal layer, inner granular layer, and polymorphic layer [8,9].

Qualitative assessments were conducted in the coronal section of cerebral cortex region and captured with TissueGnostics Axio Observer Z1 microscope digital camera at 10× and 20× (TissueGnostics GmbH, Vienna, Austria). Histology results revealed multipolar shape of pyramidal cells with large, rounded, and vesicular nuclei, whereas the granular cells exhibited large, open-face nuclei, prominent nucleoli, little cytoplasm, and neuropil with pink-stained background. Moreover, degeneration and pyknosis of neurons in the brain samples were also analyzed. The descriptions of histology examinations followed the guidelines provided by Onaolapo et al. [10].

### 2.6. Cresyl Violet (Nissl) Staining for Determination of Rough Endoplasmic Reticulum in the Cytoplasm of Neurons

About 0.5 g Cresyl violet powder (Sigma-Aldrich, St. Louis, MO, USA) was dissolved in 500 mL of distilled water alongside 1.25 mL glacial acetic acid (Sigma-Aldrich, St. Louis, MO, USA) for preparation of Cresyl violet solution (0.1%). The brain samples were placed on the slides, then washed with tap water, and dehydrated using 70% and 100% ethanol for 1 min. Next, the slices were incubated with 0.1% Cresyl violet for 15 min, washed in 70% and 100% ethanol. They were then incubated in xylene for 10 min and mounted onto slides using VectaMount permanent mounting medium (Burlingame, CA, USA). Quantitative assessments were conducted in the coronal section of cerebral cortex region and captured with TissueGnostics Axio Observer Z1 microscope digital camera at 20× (TissueGnostics GmbH, Vienna, Austria). The number of positive pyramidal Nissl-stained cells in the cerebral cortex regions and the reduction in pyramidal cell density were recorded. Randomly selected areas (200 × 200 µm^2^) from three different slides per animal were used for the study. Quantitative studies were carried out by counting number of positive pyramidal Nissl-stained cells (cell count per mm2) using ImageJ software (https://imagej.nih.gov/ij/ (accessed on 2 December 2022).

### 2.7. Western Blot Analysis of IL6, IL1β, TNFα, iNOS, NFκB, IκB, SOD2, CAT, Nrf2, Keap1, Sirt1, PGC1α, Nrf1, TFAM, Caspase-3, Bax, Bcl2, GFAP, and GAPDH Expressions

The cerebral cortex (0.3 g) was homogenized using Teflon-glass homogenizer (Qiagen^®^ Tissuelyser II, Hilden, Germany) in ice-cold 1X PBS (pH 7.4) comprising 20 M Tris-base, 7 mM NaCl, 1% Triton X-100, and 0.1% protease inhibitor. Homogenate was centrifuged (5000 rpm at 4 °C for 15 min) to remove nuclei and cell debris. After centrifugation, the supernatants were collected, and the respective protein levels determined using Pierce^®^ BCA protein assay kit (Thermo Fisher Scientific, USA). Proteins were denatured in gel-loading buffer, and approximately 20 µg proteins were resolved on the 10–15% SDS-polyacrylamide gel electrophoresis. After gel electrophoresis, the separated proteins were transferred to a polyvinylidene difluoride (PVDF) membrane (Bio-Rad Laboratories, Hercules, CA, USA).

In order to reduce background staining, membranes were incubated with 5% non-fat dry milk in Tris-buffered saline containing 1% Tween-20 (TBST) for 1 h at room temperature. Thereafter, the PVDF membranes were incubated overnight at 4 °C with various primary antibodies at specific dilutions, as highlighted in Table 1. The PVDF membranes were washed thrice with TBST, each cycle taking 10 min, and were incubated with either goat anti-rabbit horseradish peroxidase (HRP; ab205718; Abcam, Cambridge, UK) or goat anti-mouse horseradish peroxidase (HRP; ab205719; Abcam, Cambridge, UK), then visualized with an enhanced luminol-based chemiluminescent-Plus detection kit (ECL; PerkinElmer, Waltham, MA, USA). The blots were then scanned, and the images captured by a UVP^®^ digital imaging system (Analytik Jena US LLC, Beverly, MA, USA). The data were normalized against a housekeeping GAPDH protein. The number of experiments used in the quantifications was 2 or 3 replications.

### 2.8. Mitochondria DNA Copy Number Analysis

Mitochondrial DNA (mtDNA) was extracted from brain tissues as per the manufacturer’s protocol (QIAGEN; DNeasy Blood & Tissue Kits, Hilden, Germany). The concentration and quality of the extracted DNA were determined using spectrophotometer at 260 and 280 nm absorbance. The mtDNA copy number was analyzed using quantitative real-time PCR (qPCR) (Applied Biosystems, Foster City, CA, USA). Targeted mitochondrial genes included NADH-ubiquinone oxidoreductase chain 1 (ND-1) (gene accession numbers: NC_001665.2), whereas β-actin represented nuclear genes. The forward and reverse primers of β-actin were 5′-GAAATCGTGCGTGACATTAAAG-3′ and 5′-ATCGGAACCGCTCATTG-3′. The forward and reverse primers of ND-1 were 5′-TTAATTGCCATGGCCTTCCTCACC-3′ and 5′-TGGTTAGAGGGCGTATGGGTTCTT-3′ [11]. The mtDNA copy number was computed using the delta–delta cycle threshold (ΔΔCt) method.

### 2.9. Statistical Analysis

GraphPad Prism 8 (La Jolla, CA, USA) was used for statistical analyses of all the variables. Mean comparisons between groups were analyzed using a one-way analysis of variance (ANOVA) with Bonferroni’s post-test. Mean differences were considered significant at *p* < 0.05. The data are expressed as mean ± standard deviation (SD).

## 3. Results

### 3.1. Effects of Oral Administration of Aspartame on Rats’ Body and Brain Weight

As highlighted in Table 2, the body weight, body weight change from initial to final day, brain weight, and brain coefficients in female rats in the LA and HA groups were not significantly (*p* > 0.05) different when compared to the values in the control group.

### 3.2. Effects of Oral Administration of Aspartame on BDNF, COX-2, and PGE2 Levels in the Rats’ Cerebral Cortex

**Figure 1:**
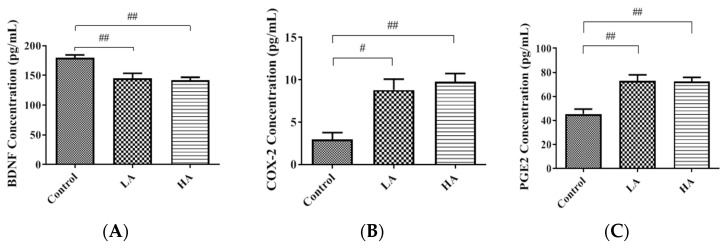
Effects of oral administration of aspartame on (**A**) BDNF, (**B**) COX-2, and (**C**) PGE_2_ levels in the rats’ cerebral cortex. Each bar represents mean ± SD; # *p* < 0.05, ## *p* < 0.01 when compared with control group by a one-way ANOVA with Bonferroni’s post-test correction. Rat groups: control diet (control), low dose of aspartame (LA), high dose of aspartame (HA).

In the current study, the brain-derived neurotrophic factor (BDNF) levels in the rat cerebral cortex were significantly reduced in the LA and HA groups when compared to those of the control group (*p* < 0.01) (Figure 1A). Cyclooxygenases-2 (COX-2) and Prostanoids (PGE_2_) are potential indicators of inflammation in the cerebral cortex. In the present study, COX-2 levels were significantly increased in the HA group (*p* < 0.05) in comparison to the control group levels (Figure 1B). Furthermore, PGE_2_ levels were elevated in both the LA and HA groups when compared with those of the control group (Figure 1C). These results imply that aspartame exposure induced neuro-inflammation in the cerebrum cortex.

### 3.3. Effects of Oral Administration of Aspartame on the Histology and Nissl Stain of the Rats’ Cerebral Cortex

**Figure 2:**
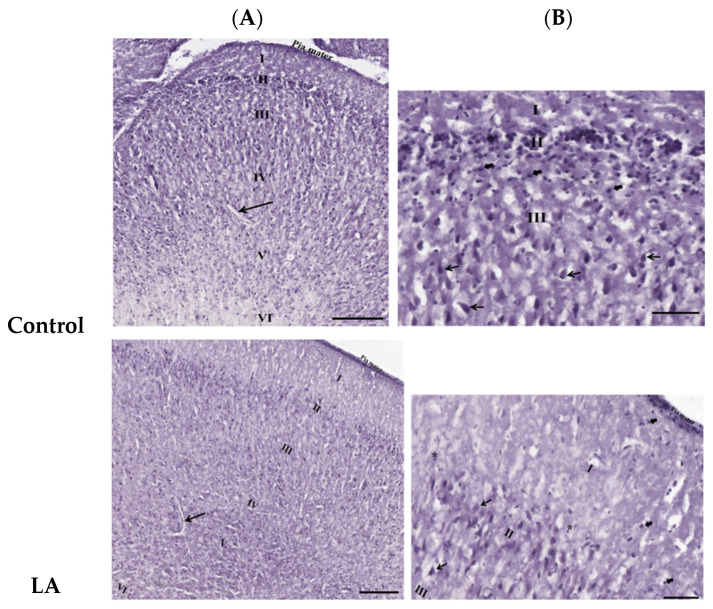

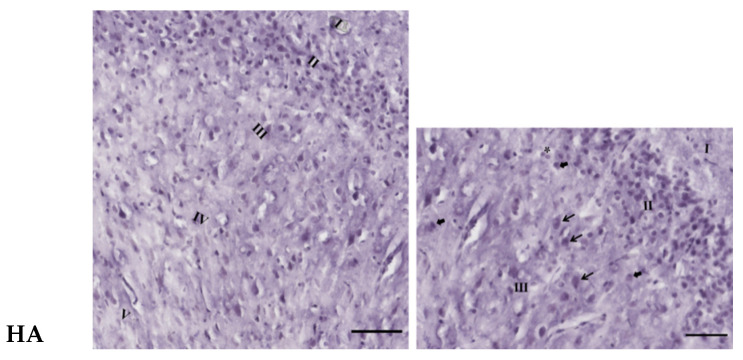
Effects of oral administration of aspartame on the morphologic changes in tissues in the rats’ cerebral cortex. H&E-stained images showing (**A**, 10×) six layers of rats’ cerebral cortex: outer molecular layer (I), outer granular layer (II), outer pyramidal layer (III), inner granular layer (IV), inner pyramidal layer (V), and polymorphic layer (VI), including the capillaries (→); (**B**, 20×) pyramidal cell (→), granule cell (*), and also neuroglial cells without properly seen cytoplasm (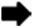). Rat groups: control diet (control), low dose of aspartame (LA), high dose of aspartame (HA).

Figure 2 shows the results from the histological examination of the cerebral cortex from hematoxylin and eosin-stained (H&E) sections. H&E is a common technique for recognizing various tissue types and the morphologic changes in tissues. Low magnification (10×) showed regular meninges, pia mater, and classical six-layered cerebral cortexes with no delineation. In accordance with protocol by Onaolapo et al. [10], the six layers observed consisted of the following: (i) a molecular layer, (ii) an external granular layer, (iii) an internal granular layer, (iv) an external pyramidal layer, (v) an internal pyramidal layer, and (vi) a multiform layer. At a magnification of 20×, we observed the layered arrangement of the cerebrum with large numbers of pyramidal cells, granule cells, and neuroglia in the control and treatment groups.

**Figure 3:**
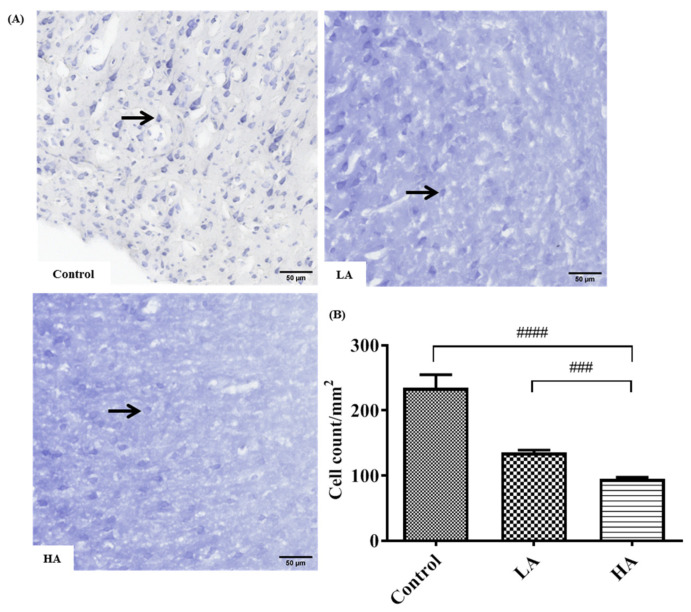
Effects of aspartame oral administration on the Nissl stain of the rats’ cerebral cortex (20×). (**A**) Control group shows deep Nissl staining in pyramidal cells. Low dose of aspartame (LA) shows mild Nissl staining in pyramidal cells. High dose of aspartame (HA) show poor Nissl staining, with least number of positive Nissl-stained pyramidal cells. (**B**) Population of Nissl-stained neurons in the cerebral cortex. Data are shown as mean ± SD; ### *p* < 0.001, #### *p* < 0.0001 when compared with control group. Black arrow refers to positive Nissl-stained pyramidal cells.

The examination of the Nissl-stained cerebral sections also showed six layers of the cerebral cortex with no delineation. In addition, the Nissl substance or rER in the cytoplasm of pyramidal cells were deeply stained in the control group. Furthermore, the number of positive Nissl-stained pyramidal cells was significantly reduced in the LA and HA groups (*p* < 0.01 and *p* < 0.0001, respectively) when compared with the levels in the control group (Figure 3).

### 3.4. Effects of Aspartame Oral Administration on Oxidative Stress and Antioxidant Marker Protein Expression of the Rats’ Cerebral Cortex

**Figure 4:**
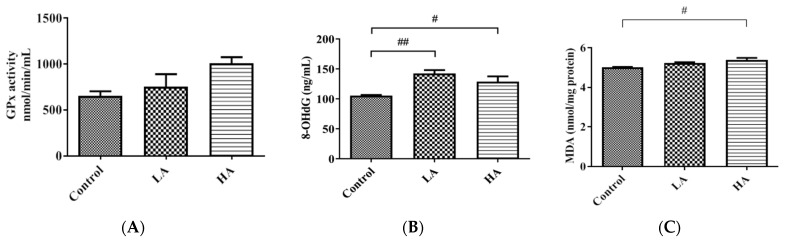
Effect of oral administration of aspartame on oxidative stress and antioxidant markers in the rats’ cerebral cortex: (**A**) GPx, (**B**) 8-OHdG, and (**C**) thiobarbituric acid reactive substance (MDA) levels in rats’ cerebral cortex in different groups. Each bar represents mean ± SD; # *p* < 0.05, ## *p* < 0.01 when compared with control group by a one-way ANOVA with Bonferroni’s post-test correction. Rat groups: control diet (control), low dose of aspartame (LA), high dose of aspartame (HA).

Although GPx levels were increased in the LA and HA groups, the levels were not significantly different from the levels in the control group (Figure 4A). Moreover, 8-OHdG levels were significantly increased in the LA (*p* < 0.01) and HA (*p* < 0.05) groups when compared with those of the control group (Figure 4B). Furthermore, the MDA levels were significantly increased in the HA group (*p* < 0.01) when compared with the control levels (Figure 4C).

### 3.5. Effects of Oral Administration of Aspartame on Pro-Inflammatory Cytokine Expression and Inflammatory Response of the Rats’ Cerebral Cortex

**Figure 5:**
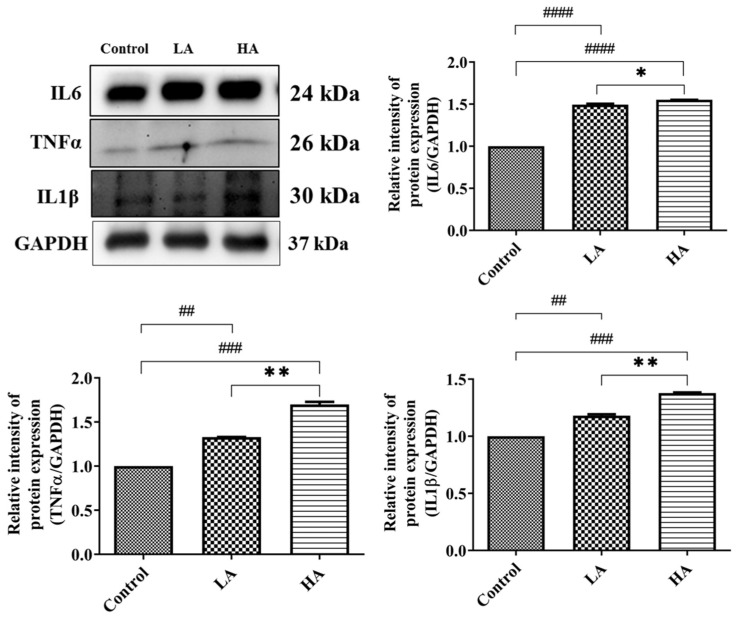
Western blot analysis of pro-inflammatory cytokine protein expression in rats’ cerebral cortex after oral administration of aspartame at different levels. Rat groups: control diet (control), low dose of aspartame (LA), high dose of aspartame (HA). Data are presented as mean ± SD. ## *p* < 0.01, ### *p* < 0.001, #### *p* < 0.0001 in comparison with the control group; * *p* < 0.05, ** *p* < 0.01 when compared between groups by a one-way ANOVA with Bonferroni’s post-test correction.

We investigated the effects of aspartame on the pro-inflammatory cytokines (TNFα, IL6, IL1β) expression levels in the cerebral cortex of the rats (Figure 5). The expression levels of TNFα were significantly elevated in the LA (*p* < 0.01) and HA groups (*p* < 0.001) when compared with those of the control group. Moreover, TNFα levels were significantly different between the LA and HA groups (*p* < 0.01). IL1β levels were significantly increased in the LA (*p* < 0.01) and HA groups (*p* < 0.001) when compared with the control group levels. Furthermore, IL6 levels were significantly increased in the LA (*p* < 0.0001) and HA groups (*p* < 0.001) when compared with the levels of the control (*p* < 0.05).

**Figure 6:**
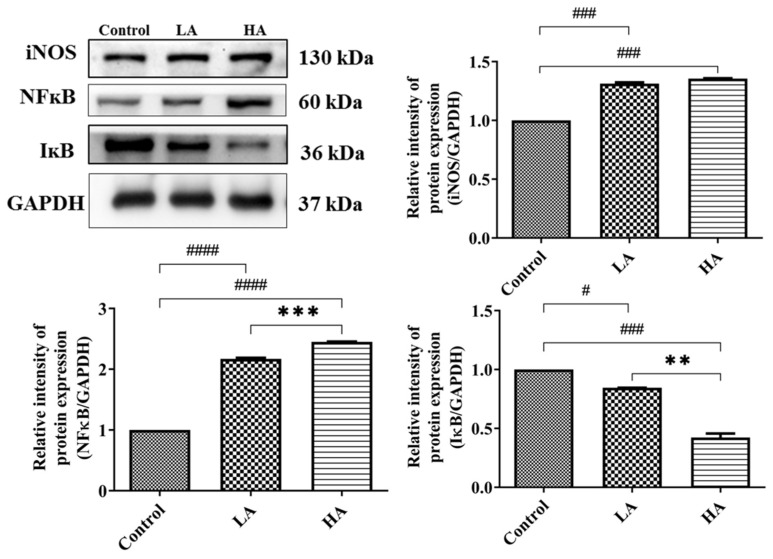
Western blot analysis of inflammatory response protein expression in rats’ cerebral cortex after oral administration of aspartame at different levels. Rat groups: control diet (control), low dose of aspartame (LA), high dose of aspartame (HA). Data are presented as mean ± SD. # *p* < 0.05, ### *p* < 0.001, #### *p* < 0.0001 in comparison with control group; ** *p* < 0.01, *** *p* < 0.001 when compared between groups by a one-way ANOVA with Bonferroni’s post-test correction.

We also investigated the effects of administration of aspartame on the inflammatory response in the cerebral cortex (Figure 6). The iNOS expression levels were up-regulated in the LA and HA groups (*p* < 0.001 and *p* < 0.001, respectively) when compared with the control group levels. Moreover, NFκB expression levels in the LA and HA groups were significantly increased (*p* < 0.0001, *p* < 0.0001, respectively) in comparison with the control group levels, and we further observed significant differences between the LA and HA groups (*p* < 0.001). On the other hand, the levels of IκB were significantly reduced in the LA (*p* < 0.05) and HA groups (*p* < 0.002) in comparison with those of the control group (*p* < 0.01). Furthermore, the levels of IκB were significantly different between the LA and HA groups (*p* < 0.01).

### 3.6. Effects of Oral Administration of Aspartame on Antioxidant Enzymes and Oxidative Biomarkers

**Figure 7:**
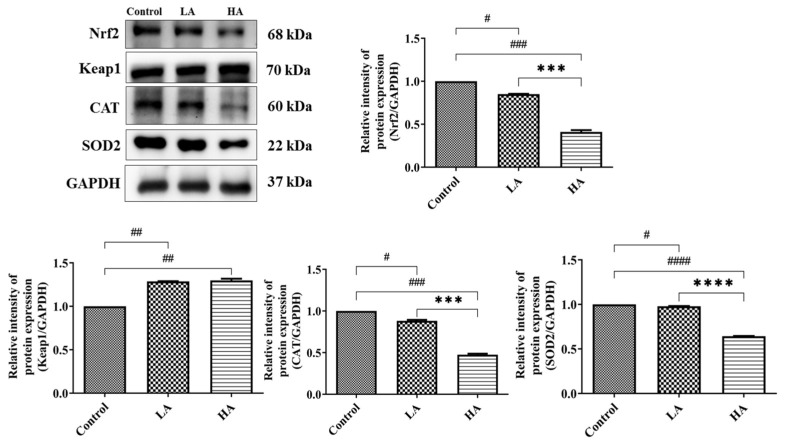
Western blot analysis of oxidative stress and antioxidant marker protein expression in rats’ cerebral cortex after oral administration of aspartame at different levels. Rat groups: control diet (control), low dose of aspartame (LA), high dose of aspartame (HA). Data are presented as mean ± SD. # *p* < 0.05, ## *p* < 0.01, ### *p* < 0.001, #### *p* < 0.0001 in comparison with control group; *** *p* < 0.001, **** *p* < 0.0001 when compared between groups by a one-way ANOVA with Bonferroni’s post-test correction.

In the present study, we examined the effects of aspartame on the expression of antioxidant enzymes (SOD2, CAT, GPx) and oxidative stress biomarkers (8-OHdG and MDA) in the cerebral cortex. The SOD2 expression levels significantly declined in both the LA group (*p* < 0.05) and the HA group (*p* < 0.0001) when compared with the levels in the control group. Moreover, SOD2 levels were significantly different between the LA and HA groups (*p* < 0.0001). CAT was significantly decreased in both the LA and HA groups (*p* < 0.05, *p* < 0.001, respectively) in comparison with the levels in the control group. CAT levels were significantly different between the LA and HA groups (*p* < 0.001) (Figure 7).

We also examined the expression levels of antioxidant markers, namely Nrf2 and Keap1 proteins. The results showed that Nrf2 levels in the LA and HA groups were significantly decreased (*p* < 0.05, *p* < 0.001) when compared with levels in the control group, and further, a significant difference was observed between the LA and HA groups (*p* < 0.001) (Figure 7). On the other hand, Keap1 protein levels were significantly upregulated in both the LA and HA groups (*p* < 0.01) in comparison with the control group levels.

### 3.7. Effects of Oral Administration of Aspartame on Mitochondrial Biogenesis Protein Expression of the Rats’ Cerebral Cortex

**Figure 8:**
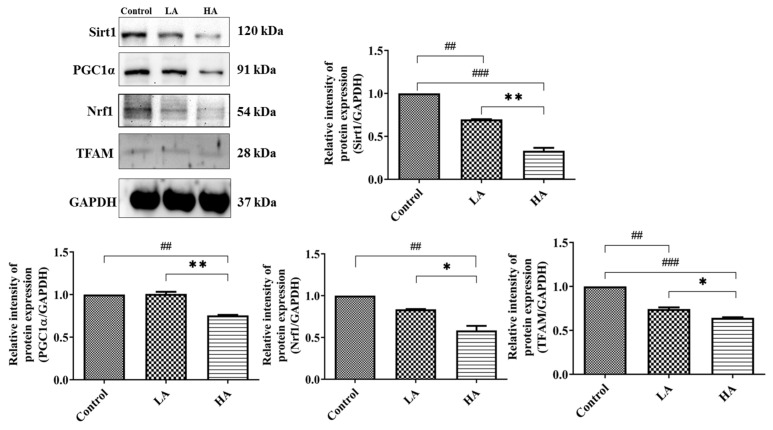
Western blot analysis of mitochondrial biogenesis-related protein expression in rats’ cerebral cortex after oral administration of aspartame at different levels. Rat groups: control diet (control), low dose of aspartame (LA), high dose of aspartame (HA). Data are presented as mean ± SD. ## *p* < 0.01, ### *p* < 0.001 in comparison with control group; * *p* < 0.05, ** *p* < 0.01 when compared between groups by a one-way ANOVA with Bonferroni’s post-test correction.

Mitochondrial dysfunction is associated with the induction of reactive oxygen species (ROS) generation in cells. In the present study, we investigated the effects of the administration of aspartame on mitochondrial biogenesis in the cerebral cortex (Figure 8). The Sirt1 expression levels significantly declined in both the LA (*p* < 0.01) and HA (*p* < 0.001) groups when compared with the control group levels. Moreover, Sirt1 levels were significantly different between the LA and HA groups (*p* < 0.01). PGC1α levels were significantly decreased only in the HA group in comparison with the control group levels (*p* < 0.01) but were significantly different between the LA and HA groups (*p* < 0.01). Similarly, Nrf1 levels were significantly decreased only in the HA group (*p* < 0.01) when compared with the control group levels, and further, the levels were also significantly different between the LA and HA groups (*p* < 0.05). Moreover, TFAM levels were markedly down-regulated in both the LA and HA groups (*p* < 0.01, *p* < 0.001, respectively) when compared with those of the control group and were also significantly different between the LA and HA groups (*p* < 0.05).

### 3.8. Effects of Aspartame Oral Administration on Apoptosis-Related Protein Expression of the Rats’ Cerebral Cortex

**Figure 9:**
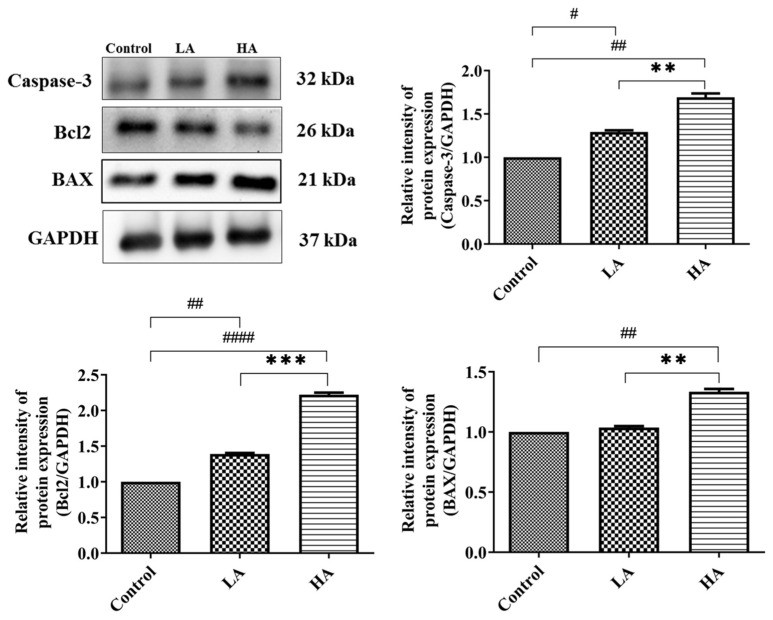
Western blot analysis of apoptosis-related protein expression in rats’ cerebral cortex after oral administration of aspartame at different levels. Rat groups: control diet (control), low dose of aspartame (LA), high dose of aspartame (HA). Data are presented as mean ± SD. # *p* < 0.05, ## *p* < 0.01, #### *p* < 0.0001 in comparison with control group; ** *p* < 0.01, *** *p* < 0.001 when compared between groups by a one-way ANOVA with Bonferroni’s post-test correction.

We investigated the effects of administration of aspartame on the apoptotic pathway in the cerebral cortex (Figure 9). The expression levels of Caspase-3 were significantly increased in the LA (*p* < 0.05) and HA groups (*p* < 0.01) in comparison to those of the control group. Similarly, Bax levels were significantly increased (*p* < 0.01) only in the HA group when compared with those of the control group. However, Bcl2 levels were significantly down-regulated in the LA (*p* < 0.01) and HA groups (*p* < 0.0001) when compared with those of the control group. Interestingly, the expression levels of Caspase-3, Bax, and Bcl2 were significantly different between the LA and HA groups (*p* < 0.05).

### 3.9. Effects of Oral Administration of Aspartame on Mitochondria DNA Copy Number of the Rats’ Cerebral Cortex

**Figure 10:**
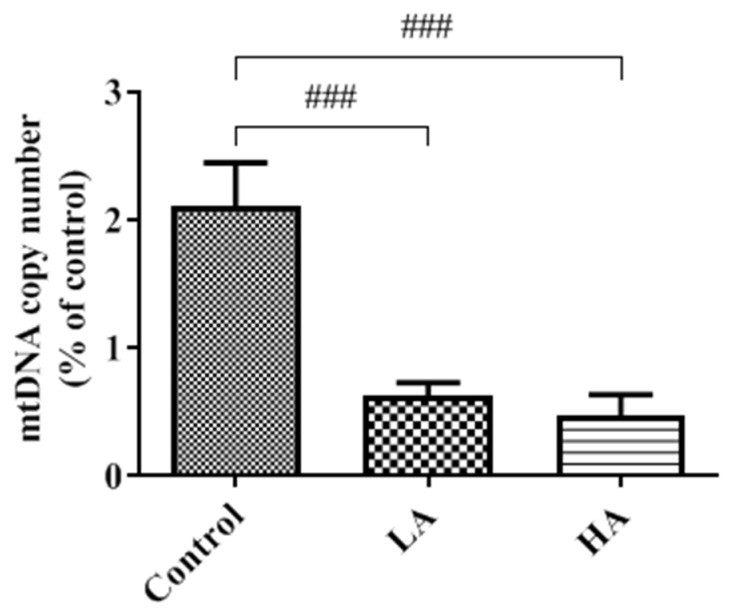
Effect of aspartame oral administration on mitochondrial DNA copy numbers in rat cerebral cortex. Each bar represents mean ± SD. Rat groups: control diet (control), low dose of aspartame (LA), high dose of aspartame (HA). Data are presented as mean ± SD. ### *p* < 0.001 when compared with control group by a one-way ANOVA with Bonferroni’s post-test correction.

mtDNA copy numbers were significantly decreased (*p* < 0.001) in both the LA and HA groups when compared with those of the control group (Figure 10).

### 3.10. Effects of Aspartame Oral Administration on GFAP Expression in the Rats’ Cerebral Cortex

**Figure 11:**
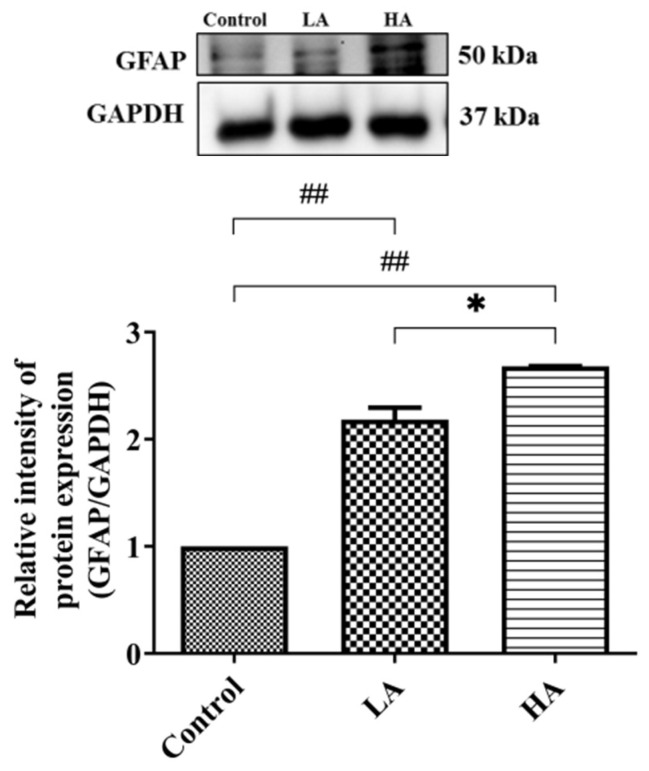
Western blot analysis of GFAP expression on rats’ cerebral cortex after oral administration of aspartame. Rat groups: control diet (control), low dose of aspartame (LA), high dose of aspartame (HA). Data are presented as mean ± SD. ## *p* < 0.01 when compared with control group, * *p* < 0.05 when compared between groups by a one-way ANOVA with Bonferroni’s post-test correction.

The expression levels of GFAP in both the LA and HA groups were significantly increased (*p* < 0.01) when compared with those of the control group and were also significantly different between the LA and HA groups (*p* < 0.05) (Figure 11).

## 4. Discussion

Overall, we evaluated the influence of oral administration of aspartame on rat cerebral cortex injury and the potential underlying molecular mechanisms, with a focus on oxidative stress, inflammation, mitochondrial biogenesis, and apoptosis factors. In the current study, the brain-derived neurotrophic factor (BDNF) levels were significantly reduced after administration of LA and HA doses for 8 weeks. These findings are in agreement with those of earlier studies that reported that long-term consumption of aspartame for 90 days [12] and 6 weeks [13] significantly decreased BDNF levels in rats’ cerebral cortex. BDNF supports cellular proliferation, cell survival, cell differentiation, branching of the dendrite, spine formation, and synaptic formation in the brain [12]. Hence, our findings suggest that chronic aspartame administration may contribute to neuronal toxicity, leading to neuronal pathogenesis or neuronal dysfunction.

In the present study, COX-2 and PGE_2_ levels were significantly increased in both LA and HA groups. These observations are in agreement with a previous report showing a significant increase in COX-2 and PGE_2_ expression levels in rats’ cerebral cortex after chronic aspartame intake (75 mg/kg bw/day) for 90 days [12]. Cyclooxygenases-2 (COX-2) are expressed in neurons as part of the inflammatory response and contribute to neurodegeneration through neuronal injury. COX-2 catalyzes the production of PGH_2_ and prostanoids (PGE_2_), which are critical in pro-inflammatory actions during inflammation. Moreover, PGE_2_ also facilitates glutamate release and excitatory synaptic transmission [12,14]. Therefore, our results imply that the administration of aspartame may have contributed to the increased inflammatory responses in the rat cerebral cortex.

We also evaluated the inflammatory response signaling pathway in order to understand the inflammation responses in the rat cerebral cortex. Nuclear factor kappa B (NFκB) is a key transcriptional protein that regulates immune and inflammatory pathways and further mediates cellular proliferation, differentiation, and apoptosis [15]. Moreover, the initiation process of inflammation involves the release of inducible NOS (iNOS), one of the NO-synthesizing enzyme isoforms [16]. Based on the findings in this study, the administration of aspartame significantly increased NFκB and iNOS expression levels in the LA and HA groups. In addition, the levels of the inhibitor of kappa B (IκB) protein were significantly reduced in the LA and HA groups. IκB is responsible for the formation of NFκB dimers in the cytoplasm [15]. These results suggest that aspartame can induce NFκB activation through the inflammatory signaling pathway and the degradation of IκB.

Taken together, the current study demonstrates that aspartame oral administration is linked to neuro-inflammation in the rats’ cerebrum cortex. NFκB is involved in the release of pro-inflammatory cytokines (TNFα, IL6, IL1β), reactive oxygen species (ROS), and inducible nitric oxide synthase (iNOS) [14]. In the current study, we found the TNFα, IL6, and IL1β expression levels were substantially elevated in both the LA and HA groups. The findings of our study corroborate those of previous studies showing significantly increased of TNFα and IL1β levels in rat brains [13] and significantly increased TNFα levels in mice brains [17] and in the rat brains [18] upon consumption of aspartame.

We further investigated the expression levels of antioxidant enzymes and found that SOD2 and CAT levels were significantly reduced, whereas MDA, 8-OHdG, and GPx levels were elevated in the LA and HA groups. Our findings are in agreement with those by Lebda et al. [4] that showed MDA and Glutathione S-Transferases (GST) levels were significantly increased while GSH, GSH-Px, SOD, and CAT declined significantly in the brains of aspartame- and soft drink-administered rats. Moreover, the subcutaneous administration of aspartame (once daily for 2 weeks) significantly elevated MDA and NO but decreased GSH levels in the mice brain [17]. In addition, previous studies reported increased SOD and NO levels in the cerebral cortex and hippocampus of rats after aspartame oral administration [10,19,20,21]. These observations imply that the early increase of SOD2 could have protected the injured region from superoxide-induced damage [22]. The accumulation of reactive oxygen species (ROS) and/or reactive nitrogen species (RNS) in biological systems is associated with oxidative damage to the structure and function of lipids, nucleic acids, proteins, and other biologically active molecules.

In the current study, we observed decreased Nrf2 and increased Keap1 expression levels in the cerebral cortex of both the LA and HA groups. Nuclear factor erythroid 2-related factor 2 (Nrf2) is found in both the cytoplasm and nucleus and is regulated by Kelch-like ECH-associated protein 1 (Keap1) [23]. Nrf2 is one of the transcriptional regulating proteins that increase cell antioxidant capacity against oxidative damage [24]. The accumulation of toxic forms of aspartame metabolites (methanol, aspartate, phenylalanine) could be the source of elevated ROS/RNS that can damage cellular proteins and DNA, including mitochondrial DNA, thus contributing to the pathogenesis of the central nervous system [22]. Therefore, from the foregoing results, we can associate the impairment of the aspartame-induced oxidative stress with the decreased Nrf2 and increased Keap1 expression levels in the rat cerebral cortex injury. It is known that 8-OHdG is a marker of oxidative damage, especially on the highly mutagenic guanine (G) nucleotides [25]. Therefore, the significantly increased 8-OHdG level in both the LA and HA groups may explain the potential link between oxidative damage in the mitochondrial DNA and mitochondrial dysfunction in the rat cerebral cortex.

In the present study, Sirt1, PGC1α, Nrf1, and TFAM expression levels in the rats’ cerebral cortex were significantly down-regulated in both the LA and HA groups. Further, the findings on this signaling pathway can be supported by the results of the mtDNA copy number. In this context, the levels of the mtDNA copy number significantly declined (*p* < 0.001) in both the LA and HA groups. The mitochondrial biogenesis pathway is regulated by transcriptional co-activators, and mitochondrial damage is generally linked to the impairment of respiration and electron transfer [26]. TFAM (mitochondrial transcription factor A) protein increases mtDNA replication and transcription, including protecting mtDNA from ROS attack [27]. Previous studies have reported the potential link between aspartame consumption, mitochondrial disorder-induced impaired ovarian function, and infertility risk in rats [11]. In the current study, the combined effects of down-regulated Sirt1, PGC1α, Nrf1, and TFAM expression levels as well as the decreased mtDNA copy number arising from the long-term consumption of aspartame can be attributed to the increased mitochondrial ROS generation.

We further determined the effects of aspartame consumption on the apoptosis pathway and found that expression levels of Caspase-3 and Bax were significantly increased, whereas Bcl2 (anti-apoptotic proteins) expression levels were significantly reduced in the LA and HA groups. The observations from our study are consistent with those of previous reports indicating a marked increase in Bax and Caspase-3 and decreased Bcl2 mRNA expression in the cerebral cortex, cerebellum, hippocampus, and hypothalamus regions of aspartame-methotrexate-treated rats [28]. In addition, Onaolapo et al. [21] reported significantly increased Caspase-3 expression in rats’ cerebellar cortex and pons after administration of a high dose of aspartame (160 mg/kg). Elsewhere, mRNA expression levels of Bax, Caspase-3, P27, and Mdm2 were up-regulated in the brains of aspartame- and soft drink-treated rats, whereas Bcl2 mRNA expressions were down-regulated [4]. In the present study, aspartame induced apoptosis in the cerebrum cortex through an increased expression of Caspase-3 and Bax and an inhibition of anti-apoptotic protein (Bcl2) expression.

Histological results showed a six-layered cerebral cortex with no delineation and the presence of pyramidal cells, granule cells, and neuroglia in the control and all treatment groups. On the other hand, Nissl staining revealed poor characteristics and a reduced number of positive pyramidal cells in the LA and HA groups. These findings corroborate those from previous reports [10,19,20,21], where Nissl-stained pyramidal cells in the cerebral cortex and hippocampus exhibited poor characteristics after the administration of 40, 80, and 160 mg/kg of aspartame. A decline in the number of Nissl-stained positive cells is strongly associated with marked mitochondrial dysfunction and could be associated with the increased levels of apoptosis pathway factors that were observed elsewhere in this study. In the current study, glial fibrillary acidic protein (GFAP) expression levels were increased in both the LA and HA groups. These results are in agreement with those from a previous study that showed increased GFAP-positive levels in the rat cerebral cortex after the administration of 160 and 320 mg/kg aspartame [10,19]. Our findings imply that aspartame administration can modulate oxidative stress-associated cell-signaling pathways in both neuron and neuroglial cells in the rat cerebral cortex. Taken together, the findings of this study demonstrate that although aspartame has not been conclusively linked with any serious health effects, people with nerve-related disorders should be careful when consuming it.

**Figure 12:**
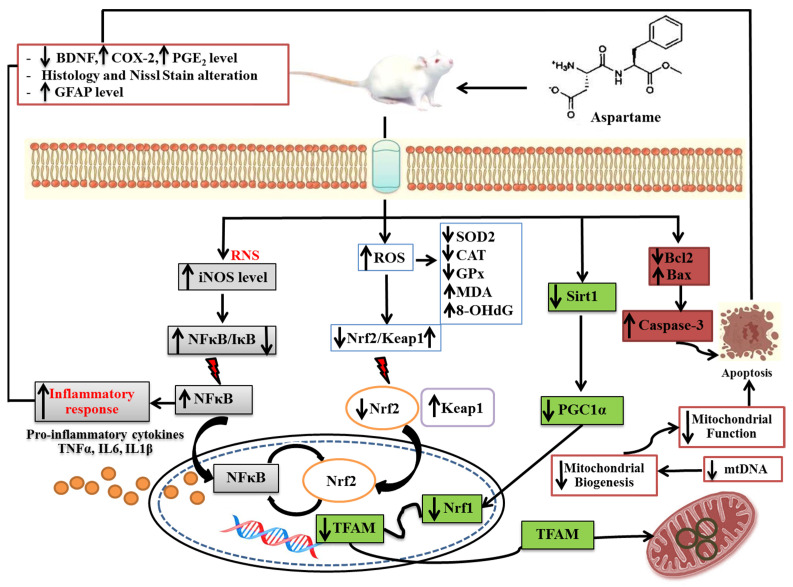
Schematic illustration of aspartame-induced rats’ cerebral cortex injury. The neurotoxicity effects could be regulated through four mechanisms: enhanced inflammation, enhanced oxidant stress, decreased mitochondrial biogenesis, and apoptosis pathways. These four mechanisms could directly or indirectly affect the survival of pyramidal cells and granular cells, including astrocyte glial cells of cerebral cortex.

To the best of our knowledge, the present study demonstrates for the first time a potential novel association between aspartame-induced rat cerebral cortex injury and an integration of various underlying molecular pathways, including oxidative stress, mitochondrial dysfunction, inflammation, and apoptosis in the cerebral cortex of rats, as illustrated in Figure 12.

## 5. Conclusions

The findings of the study demonstrate the potential effects of oral aspartame consumption on the rat cerebral cortex injury (reduced survival of pyramidal cells and astrocyte glial cells) through various potential underlying molecular mechanisms, including reduced levels of BDNF, enhanced oxidative stress, inhibited antioxidant capacity, inhibited mitochondrial biogenesis and enhanced apoptosis-related protein expression (Bax, Caspase-3), and elevated levels of pro-inflammatory cytokines and inflammatory responses (PGE_2_, COX-2).

## Figures and Tables

**Table 1:** List of primary antibodies used in Western blotting.

Primary Antibody	Host	Dilution	Molecular Weight (kD)	Catalog Number	Source
anti-IL6	rabbit	1:1000	24	DF6087	Affinity Biosciences, Cincinnati, OH, USA
anti-IL1β	rabbit	1:1000	30	AF5103	Affinity Biosciences, Cincinnati, OH, USA
anti-TNFα	mouse	1:500	26	60291-1-Ig	Proteintech, Rosemont, IL, USA
anti-iNOS	rabbit	1:1000	130	AF0199	Affinity Biosciences, Cincinnati, OH, USA
anti-NFκB	rabbit	1:1000	60	622601	Biolegend, San Diego, CA, USA
anti-IκB	rabbit	1:1000	36	10268-1-AP	Proteintech, Rosemont, IL, USA
anti-SOD2	rabbit	1:1000	22	13194	Cell Signaling, Danvers, MA, USA
anti-CAT	mouse	1:1000	60	863301	Biolegend, San Diego, CA, USA
anti-Nrf2	rabbit	1:1000	68	16396-1-AP	Proteintech, Rosemont, IL, USA
anti-Keap1	rabbit	1:1000	70	10503-2-AP	Proteintech, Rosemont, IL, USA
anti-Sirt1	mouse	1:1000	120	8469	Cell Signaling, Danvers, MA, USA
anti-PGC1ɑ	rabbit	1:1000	91	NBP1-04676	Novus Biologicals, Centennial, CO, USA
anti-Nrf1	rabbit	1:1000	54	AB175932	Abcam, Cambridge, UK
anti-TFAM	rabbit	1:1000	28	AB131607	Abcam, Cambridge, UK
anti-Caspase-3	rabbit	1:1000	32	19677-1-AP	Proteintech, Rosemont, IL, USA
anti-Bax	rabbit	1:1000	21	50599-2-Ig	Proteintech, Rosemont, IL, USA
anti-Bcl2	rabbit	1:1000	26	26593-1-AP	Proteintech, Rosemont, IL, USA
anti-GFAP	rabbit	1:1000	50	DF6040	Affinity Biosciences, Cincinnati, OH, USA
anti-GAPDH	rabbit	1:1000	37	2118	Novus Biologicals, Centennial, CO, USA

**Table 2:** Effect of aspartame oral administration on body weight, brain weight, and brain coefficients in rats.

Group	Body Weight (g)		Weight Change (g)	Brain Weight (mg)	Brain Coefficients (mg/100 g BW)
	Initial	Final			
Control	211.90 ± 5.93	295.30 ± 13.60	83.40 ± 11.48	1.90 ± 0.08	0.6431 ± 0.0380
LA	211.21 ± 8.60	298.71 ± 20.12	87.50 ± 15.57	2.00 ± 0.06	0.6724 ± 0.0363
HA	206.43 ± 8.50	285.79 ± 20.40	79.36 ± 13.56	2.08 ± 0.08	0.6959 ± 0.0395

Values are expressed as mean ± SD (control: *n* = 5, LA: *n* = 7, HA: *n* = 7). All values in the same column are not significantly different, *p* > 0.05 (one-way ANOVA followed by Bonferroni’s multiple comparison test). Rat groups: control diet (control), low dose of aspartame (LA), high dose of aspartame (HA).

## Data Availability

Data are contained within the article.
